# Performance Monitoring and Correct Response Significance in Conscientious Individuals

**DOI:** 10.3389/fnhum.2019.00239

**Published:** 2019-07-10

**Authors:** Mike F. Imhof, Jascha Rüsseler

**Affiliations:** Department of Psychology, University of Bamberg, Bamberg, Germany

**Keywords:** error-related negativity, correct-response negativity, CRN, conscientiousness, five factor model, response monitoring, motivational salience, task engagement

## Abstract

There is sufficient evidence to believe that variations in the error-related negativity (ERN) are linked to dispositional characteristics in individuals. However, explanations of individual differences in the amplitude of the ERN cannot be derived from functional theories of the ERN. The ERN has a counterpart that occurs after correct responses (correct-response negativity, CRN). Based on the assumption that ERN and CRN reflect an identical cognitive process, variations in CRN might be associated with dispositional characteristics as well. Higher CRN amplitudes have been found to reflect task engagement. In the present study, a simple-choice-reaction task was used to investigate ERN and CRN amplitudes in relation to their score on a conscientiousness scale. The task consisted of a simple rule that required pressing the left or right key when a circle or square appeared, respectively. During alternative conditions that occur infrequently, participants were instructed to violate or reverse the previously established response rules. Smaller ΔERN amplitudes (manifested in almost equal CRN and ERN amplitudes) and a tendency of better task performance from participants scoring high on the conscientiousness scale might indicate a greater focus on the task and higher motivation of responding correctly. In addition, higher Pc amplitudes directly following the CRN indicated that the response monitoring system of less conscientious participants showed a higher disengagement. The role of individual differences in CRN amplitude should be studied in future experiments on performance monitoring.

## Introduction

### Error-Related Negativity

Error-related negativity (ERN; Gehring et al., 1990, 1993) which is also referred to as error negativity (Ne; Falkenstein et al., 1991) is a response-locked negative deflection in the event-related potential (ERP) that results from the commission of an error. It occurs within 100 ms following erroneous responses and its scalp distribution is maximal at midline frontocentral scalp locations. The ERN most likely originates in the anterior cingulate cortex (ACC; Gehring et al., 1993; Dehaene et al., 1994; van Veen and Carter, 2002). The ERN is followed by the error positivity (Pe), a positive deflection that is usually peaking around 150–350 ms after response onset at parietal scalp locations (Falkenstein et al., 1991; Overbeek et al., 2005). It is believed to reflect error awareness (Steinhauser and Yeung, 2010) or confidence about response correctness (Boldt and Yeung, 2015).

### Correct-Response Negativity

Correct responses often trigger a negativity that has an equal latency in the response-locked waveform as the ERN but with a smaller amplitude (Ford, 1999; Falkenstein et al., 2000; Vidal et al., 2000). Both components have a similar topography (Luu et al., 2000b; Vidal et al., 2000) and presumably the same source in the ACC (Roger et al., 2010). Their waveforms are strikingly similar, particularly in individual subject data (Swick and Turken, 2002). The component is called correct-response negativity (CRN; Ford, 1999) due to the similarity with the ERN. However, ERN is much larger on error trials than CRN on correct trials. On a flanker task where e.g., stimuli consist of arrows, correct responses to incongruent stimuli (i.e., target arrow pointing to a different direction than the surrounding arrows, e.g., “<<><<”) elicit a larger CRN than correct responses to congruent stimuli (i.e., central and surrounding arrows pointing to the same direction, e.g., “<<<<<”; Bartholow et al., 2005). CRN is largest when the presented trial type is different than expected (i.e., when an incongruent trial appeared while a congruent trial was expected) showing that changes in CRN amplitude from trial to trial not only reflect response conflict but also strategic processes. According to control models (Kerns et al., 2004; Ridderinkhof et al., 2004) either type of conflict is sufficient to elicit a signal from the ACC to the prefrontal cortex (PFC) indicating that an increase in attention is required.

It has been observed that an inverse relationship between the probability of committing an error and the magnitude of the CRN exists (Allain et al., 2004). Larger CRN amplitudes may have a preventive function against errors and the magnitude of the CRN may indicate the degree of engagement of response monitoring on correct trials (Simons, 2010).

Due to their similarities some authors assume that ERN and CRN are not distinct components but reflect an identical cognitive control process during response monitoring that is specifically enhanced on error trials (Suchan et al., 2007; Burle et al., 2008; Meckler et al., 2011). However, there is also evidence that ERN and CRN are distinct, i.e., reflecting different processes (Yordanova et al., 2004; Vocat et al., 2008). Another perspective is that both components reflect the combined activity of two underlying processes each reflecting different aspects of performance monitoring (Endrass et al., 2012).

### Functional Theories of the ERN

Several theories explaining the functional significance of the ERN have been proposed. The conflict monitoring theory assumes a response conflict (a concurrent activation of multiple competing responses) that is triggered in typical choice tasks and which signals the need for increased cognitive control (Carter et al., 1998; Botvinick et al., 2001; Yeung et al., 2004). However, there are some studies that question the conflict monitoring approach (Carbonnell and Falkenstein, 2006; Masaki et al., 2007; Burle et al., 2008). According to reinforcement learning theory (RL-ERN), an error signal is produced by monitoring mechanisms and is triggered when events occur that violate expectations. In this case, an error signal is conveyed to the ACC by the midbrain dopaminergic system. In the ACC the signal is used to improve task performance by influencing how control over the motor system is allocated to different competing systems in the brain (Holroyd and Coles, 2002).

According to RL-ERN as well as conflict monitoring accounts, variation in the amplitude of the ERN is associated with current behavior and the ERN itself serves to form subsequent behavior. Adaptive responses to errors, like post-error slowing (PES), are behavioral adjustments to improve task performance (Holroyd and Coles, 2002; Holroyd et al., 2005). However, the relationship between variation in the ERN and behavioral measures still remains unclear because multiple instances exist in which variation in the ERN occurs although behavioral differences are absent (for a review, see Weinberg et al., 2012). While the mechanisms responsible for the generation of the ERN appear to be similar across individuals, there is evidence that the ERN amplitude is moderated by situational, motivational and affective processes (Wiswede et al., 2009), as well as more stable traits that differ inter-individually (Pailing and Segalowitz, 2004). Neither RL-ERN nor conflict monitoring theory adequately explains the individual differences that have been observed in the ERN.

### ERN/CRN and Individual Differences

The ERN amplitude seems to be affected by motivational salience. It is enhanced when error significance is emphasized, either through task instructions that stress accuracy over speed (Gehring et al., 1993; Falkenstein et al., 2000), external performance evaluation (Hajcak et al., 2005a; Kim et al., 2005), or incentives (Pailing and Segalowitz, 2004; Hajcak et al., 2005a; Chiu and Deldin, 2007; Ganushchak and Schiller, 2008; Endrass et al., 2010; Riesel et al., 2012).

Amplitude of the ERN also appears to be increased in individuals who experience errors as more aversive than other individuals. A recent meta-analysis suggests that a larger ERN is related to anxiety, specifically apprehension/worry, while a larger CRN is not reliably associated with anxiety (Moser et al., 2013). Enhanced ERN amplitude has been also observed in individuals with obsessive-compulsive disorder (OCD; e.g., Gehring et al., 2000; Johannes et al., 2001; Ruchsow et al., 2005; Endrass et al., 2008, 2010; Riesel et al., 2011). ERN is also positively related to symptom severity in OCD patients (Gehring et al., 2000). Healthy individuals scoring high in negative affect and emotionality (e.g., fear and anxiety; Luu et al., 2000a) or with increased scores on the Behavioral Inhibition System scale are also characterized by enhanced ERN amplitudes (Boksem et al., 2006). During tasks that penalize error responses, reduced ERN amplitudes are associated with low scores on trait socialization (Dikman and Allen, 2000) indicating a smaller sensitivity to punishment.

ERN amplitude has also been shown to vary with personality dispositions. Motivational manipulations that impact ERN amplitude may be moderated by personality traits (Pailing and Segalowitz, 2004). As an example, Olvet and Hajcak (2011) observed that the impact of sad mood on ERN amplitude is moderated by neuroticism.

In a recent review article, Weinberg et al. (2012) assumed that a reduced ERN, related to externalizing traits and psychopathology, may reflect motivational disengagement, disinhibition, and decreased conscientiousness. Furthermore, enhanced ERN may reflect characteristics that are common to anxiety disorders such as perfectionism, concern over errors, negative affect and increased intolerance of uncertainty. These characteristics may have a strengthening effect on error significance. Poorer performance and smaller ERN may, therefore, both be caused by task disengagement and motivational deficits, whereas larger ERNs may be linked to increased motivational significance of errors.

To date, the relationship of individual differences and CRN amplitude has not been explored in detail. There is evidence that ERN and CRN amplitudes are larger in participants with obsessive-compulsive characteristics (Hajcak and Simons, 2002) and bilinguals (Kałamała et al., 2018) indicating differences in task strategy and higher task engagement.

Additionally, while there is a huge body of research concerned with the relationship of psychopathology and other individual differences with ERN, there are only a few studies examining the role of personality traits, especially conscientiousness, in the generation of the ERN amplitude. Hill et al. (2016) found that at low levels of conscientiousness, negative urgency (i.e., impulsivity in the context of negative affect) had a positive impact on the magnitude of the ERN. Pailing and Segalowitz (2004) observed that individuals higher on conscientiousness were characterized by smaller motivation-related changes in the ERN across monetary incentives.

### The Present Study

In this study, we wanted to examine whether conscientiousness is related to an increased motivational salience of an error and whether individuals scoring high on a conscientiousness scale display a stronger task engagement than individuals with low levels of conscientiousness. Increased motivational salience is associated with enhanced ERN and accompanied with increased error significance and stronger task engagement (Weinberg et al., 2012). Individuals scoring high on conscientiousness may thus have a larger ERN or even a larger CRN amplitude.

Furthermore, we wanted to investigate the relationship of rule violations and medial-frontal response-locked ERPs. The ERN appears to elicit after slips during unwilled actions but not after mistakes during willed actions (Stemmer et al., 2001). However, violating a rule may evoke a higher response conflict in individuals (Pfister et al., 2016b; Wirth et al., 2016; Jusyte et al., 2017) and stronger response conflict is associated with a larger magnitude of ERN and CRN (Bartholow et al., 2005). According to conflict monitoring accounts, violating a rule could be reflected by variations in the magnitude of ERN and CRN. We wanted to determine whether there is an enhanced CRN when violating a rule compared to rule compliant behavior. Especially participants with high levels of conscientiousness may have a higher response conflict when they are forced to violate a rule and may thus have an even more enhanced CRN during rule violations.

In the present study, a choice reaction task establishing a rule with clear S-R contingency was designed. Participants had to respond to a circle by pressing the left key, to a square by pressing the right key. In two other conditions participants had to either violate or reverse this rule. It is particularly significant that the S-R mapping was the same in both alternative conditions. The only way to follow the instructions and violate the rule was doing the opposite as in the standard condition, i.e., pressing the left key when a square occurred, pressing the right key when a circle appeared. The alternative conditions allowed us to compare rule-consistent with rule-violation behavior. Importantly, both alternative conditions required the same response. If there was a behavioral or electrophysiological effect of rule violations it should have an impact on the difference in response times (RTs) and error rates and the response-locked medial-frontal ERP. To examine the relationship of conscientiousness with response monitoring, we administered the conscientiousness scale of the NEO Five Factor Inventory (NEO-FFI; Borkenau and Ostendorf, 2008) to our sample.

We expected generally prolonged RTs and higher error rates for rule violations compared to rule-compliant behavior. The medial-frontal response-locked ERP during rule violations in both erroneous and correct responses presumably show an accentuated negativity in a time window of 0–100 ms (corresponding to ERN and CRN) compared to the rule-based conditions. Additionally, we expected participants scoring high on the conscientiousness scale being faster in responses and committing fewer errors reflecting higher task engagement and greater concern over errors. At the same time, participants with high conscientiousness values were assumed to show prolonged PES. We also assumed that individual differences in the medial-frontal response-locked ERP after erroneous and correct responses were associated with conscientiousness. More specifically, ERN and CRN measured at frontocentral electrodes were assumed to be more pronounced in individuals with high levels of conscientiousness.

## Materials and Methods

### Participants

Forty-six participants (42 females, 2 left-handed) between ages of 18 and 47 years (*M* = 22.6, *SD* = 8.5), mainly undergraduate university students were recruited and received 2.5-h course credit. All participants reported that they were free of neurological disorders and had normal or corrected-to-normal visual ability. One participant had to be excluded from EEG analysis due to current use of psychoactive medication. We had to exclude another 18 participants (39%) from all analyses due to commission of too few errors (<5) in either of both alternative instruction conditions Rule violation (RV) and Rule reversal (RR). This massive shrinkage of our sample may have been due to a rather low level of task difficulty while at the same time the absolute number of trials in some conditions was very low (120). All participants were naive concerning the hypotheses underlying the experiment and had signed a consent form prior to participation in the study. The study was conducted in accordance with the Declaration of Helsinki. The ethics committee of the University of Bamberg approved the study protocol.

### Materials

#### Questionnaires

The NEO-FFI (Borkenau and Ostendorf, 2008, original version by Costa and McCrae, 1992) was used. It is a shorter version of the Revised NEO Personality Inventory (NEO-PI-R; Costa and McCrae, 1992) with 60 items derived from the original 240 items. The five domains assessed with 12 items each by the NEO-FFI are conscientiousness, extraversion, agreeableness, neuroticism, and openness. Each item is rated on a 5-point scale.

#### Stimuli and Apparatus

Stimuli were displayed on a 24″ screen with a resolution of 1,600 × 900 pixels and participants had an average viewing distance of 65 cm to screen. A button box (Cedrus RB-830, San Pedro, CA, USA) served as response device. Stimuli consisted of a green circle with a diameter of 28 mm or a green square that measured 28 × 28 mm. This corresponds to a display size of 2.5° of visual angle for the stimuli and 9.0° × 3.0° of visual angle for instructions. There was one button for each left and right responses. Participants were asked to respond bimanually and to use the index finger of each hand to press the buttons. The randomized presentation of stimuli and instructions was controlled by Presentation Version 16 (Neurobehavioral Systems Inc., Albany, CA, USA). The correct response was determined by the target stimulus and one of three instructions. Participants were instructed to respond in accordance with the overall rule that required to press the left button when the stimulus was a circle and to press the right button when the stimulus was a square.

### Procedure

**Figure 1:**
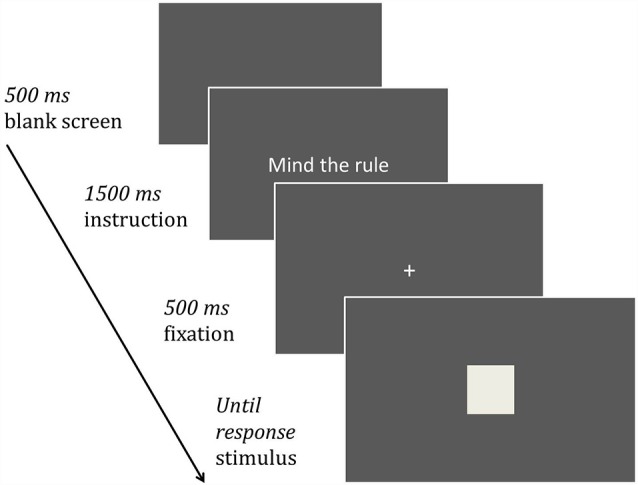
Procedure of a trial. After the presentation of a blank screen for 500 ms, the participant is instructed by a short text for 1,500 ms. Then the presentation of a fixation cross for 500 ms follows whereafter the target stimulus is presented until the participant responds.

The trial procedure is illustrated in Figure 1. Trials started with a blank screen (500 ms), followed by the presentation of an instruction (1,500 ms) according to one of the three conditions. After that, a fixation cross was presented (500 ms) and the target stimulus (circle or square) followed. The target remained on screen until a response was given. Possible response types were pressing the left or the right button to the target that was either a circle or a square.

The experimental conditions were administered as follows. Depending on the condition, instruction and correct answers were:

- Standard (STD): “Mind the rules,” circle—left, square—right.
- Rule violation (RV): “Commit an error,” circle—right, square—left.
- Rule reversal (RR): “Reverse the rules,” circle—right, square—left.

The experiment consisted of 30 blocks of 20 trials each (600 trials in total). The standard condition included 60% of the trials whereas the other two tasks comprised 20% each. All trials were presented in a randomized order across the experiment. The visual stimuli types (circles and squares, 300 each) were equally distributed over all conditions. The experimental manipulation was implemented in terms of different instructions across trials. Before the experiment started, participants had to practice the mapping rules in a training phase of 20 trials. During the experiment, participants determined the length of breaks between blocks themselves.

### EEG Recording and Data Analysis

EEG was recorded by means of an EasyCap (EASYCAP GmbH, Germany) equipped with sintered Ag-AgCl-electrodes. For the recording we used electrodes F1, F2 C3, C4, CP1, CP2, CP5, CP6, Cz, F3, F4, F7, F8, FC5, FC6, FCz, Fz, O1, O2, P3, P4, P7, P8, Pz, T7, T8, TP10, TP9 placed according to the 10–20-system (Jasper, 1958). In addition, two electrodes were placed above the left and right mastoid. The electrode AFz was used as ground and an electrode on the nose tip as reference. However, only recorded data from electrodes FCz and Pz were used for statistical analysis. The EEG signal was amplified by a BrainAmp amplifier (Brain Products GmbH, Germany) with a sampling rate of 250 Hz and 16 bit/channel. Using a band-pass filter of 0.01 Hz to 30 Hz the signal was filtered to eliminate skin conductance and muscle activity interference. Additionally, a notch filter with 50 Hz was used. All impedances were maintained below 10 kO during the entire recording procedure. After the experiment, EEG data were further prepared in BrainVision Analyzer 2.0.1 (Brain Products GmbH, Germany). Only trials with responses between 200 ms and 1,500 ms after stimulus presentation were considered for analysis. Before ERP data analysis, all trials containing artifacts of eye movements were corrected using a blind component separation (Joyce et al., 2004), which has been shown to be superior to other artifact correction procedures (Kierkels et al., 2006).

For purpose of data analysis, we averaged waveforms with a 100 ms pre-response baseline and extended the epoch to 600 ms post-response. A baseline correction was applied to 100 ms pre-response interval. Epochs with voltage steps of 20 μV/4 ms or differences of 300 μV in an interval of 150 ms on each channel were rejected from further data analysis.

We analyzed the error-related components ERN and Pe and the component CRN which is the counterpart of ERN in correct responses. The ERN/CRN is observed as the maximum amplitude of the negative deflection within the first 100 ms after (incorrect) response onset over fronto-central electrodes along the midline (electrodes Fz and FCz). The ERN/CRN was measured in response-locked ERP averages. The Pe follows the ERN and is a positive deflection, usually peaking around 150–350 ms after response onset. Its maximum amplitude is observed over centro-parietal electrodes along the midline (electrodes Cz and Pz). Depending on the electrophysiological properties of the present ERP dataset, we defined the ERN/CRN as the mean amplitude during 20–70 ms post-response interval at electrode FCz. The Pe/Pc was calculated as the mean amplitude during 200–350 ms interval following response onset at electrode Cz. Table 1 shows the mean trial numbers that were included in the analysis due to the overall low error rates in this experiment.

**Table 1:** Mean number of trials for each combination of instruction condition and response type that were included in statistical analyses.

Condition	Response type	
	Correct	Error
Standard	337.18	10.62
Rule violation	107.89	7.19
Rule reversal	106.86	8.00

For all RT analyses, data were corrected for outliers by removing trials with RTs that deviated more than 2.5 SDs from the mean RT of each participant and condition.

Typically, RTs after erroneous responses are slower compared to RTs after correct responses (PES). We defined the measure of PES as follows:

$${\text{PES=RT}}_{\text{Correct}(\text{STD})\to \text{Correct}(\text{STD})}{\text{−RT}}_{\text{Error}(\text{STD})\to \text{Correct}(\text{STD)}}$$

We assessed effects of rule condition (STD vs. RV vs. RR), conscientiousness (continuous) and accuracy (error vs. correct) as well as their interactions on response-locked ERPs *via* linear mixed-effects regression using the lme4 package in R (Version 3.5.1; Bates et al., 2014). In addition, we analyzed effects of rule condition (STD vs. RV vs. RR) and conscientiousness (continuous) including their interactions on the behavioral measures error rate and RT also by means of linear mixed-effects regression. Linear mixed-effects regression provides several important advantages over traditional methods such as repeated-measures analysis of variance (ANOVA); it allows us to include conscientiousness as a continuous variable and rule condition as repeated-measures variable. We obtained *p*-values for all linear mixed-effects models using the ANOVA function of the lmerTest package (Kuznetsova et al., 2017) with a Kenward-Roger approximation of degrees of freedom. As random effects, we had intercepts for subjects, as well as by-subject random slopes for the effect of conscientiousness and rule condition. Furthermore, mean values of PES were analyzed *via* linear regression with regressing PES onto conscientiousness.

## Results

### NEO-FFI Scales

**Figure 2:**
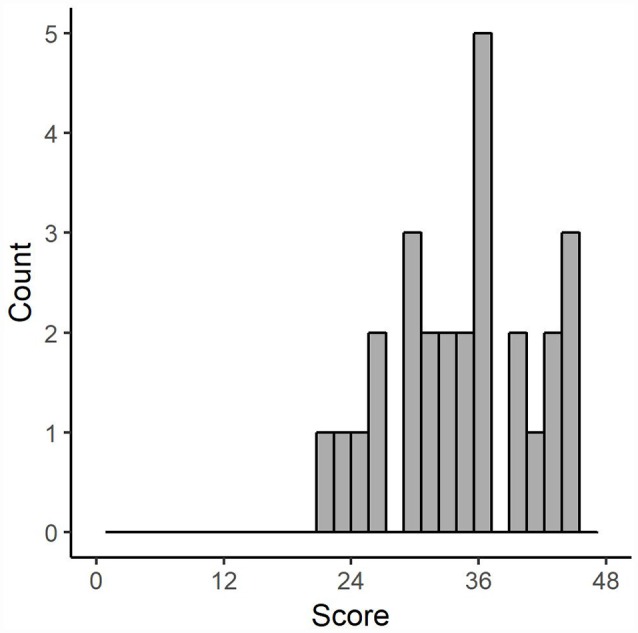
Distribution of conscientiousness scores obtained from our sample.

Sufficient ranges in conscientiousness (*Min* = 22, *Max* = 48, *MD* = 36, *M* = 35.47, *SD* = 6.90) obtained from our sample with possible ranges of 0–48 were achieved. Figure 2 shows the distribution of conscientiousness scores. Descriptive statistics and internal consistency values are provided in Table 2, intercorrelations among the personality scales in Table 3. A negative relationship between openness and conscientiousness, *r* = −0.32 (*p* < 0.05) is the only significant relationship among the measured personality scales.

**Table 2:** Descriptive statistics (Mean, SD) and internal consistency (cronbach’s alpha) for personality traits measured by NEO-Five Factor Inventory (NEO-FFI).

Personality scale	M (SD)	*α*
Neuroticism	22.49 (7.57)	0.88
Extraversion	28.82 (5.45)	0.73
Openess	32.98 (6.42)	0.81
Agreeableness	34.16 (6.43)	0.84
Conscientiousness	35.47 (6.90)	0.86

**Table 3:** Intercorrelations among personality traits measured by NEO-FFI.

Personality scale	EV	ON	AA	CS
Neuroticism (NT)	−0.20	0.09	−0.24	0.01
Extraversion (EV)	-	0.05	0.23	0.07
Openness (ON)		-	−0.01	−0.32*
Agreeableness (AA)			-	−0.12
Conscientiousness (CS)				-

Notes. n = 28. *p < 0.05.

### Behavioral Data

**Figure 3:**
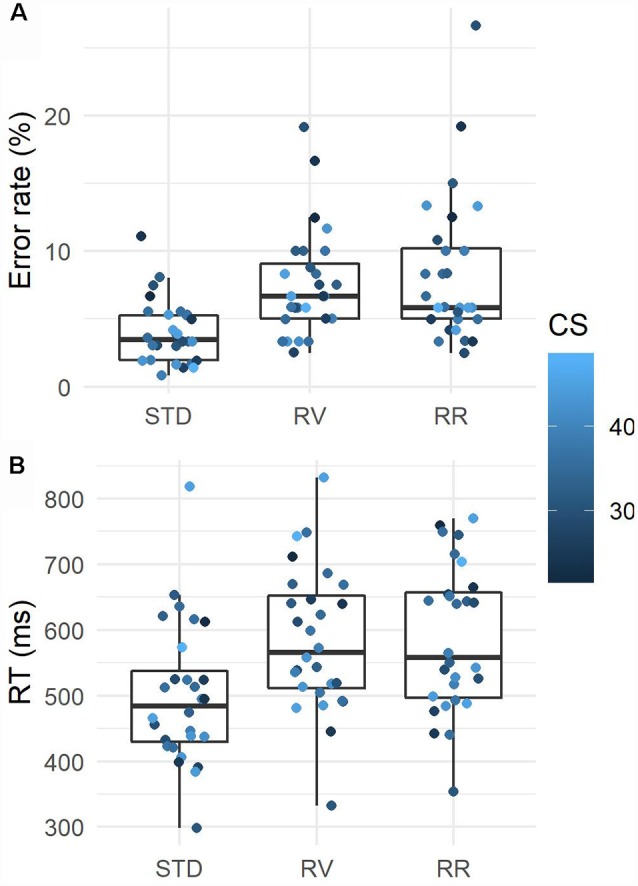
Mean error rates **(A)** and response times (RTs; **B**) for each instruction condition, standard (STD), rule violation (RV), and rule reversal (RR). Scatter plots display the performance as a function of conscientiousness (CS). See text for details.

To analyze the influence of rule condition and conscientiousness on behavioral performance, we performed two linear mixed-effects regression analyses for both error rate and RT (see Figures 3A,B). As fixed effects for each analysis, we entered the rule condition and conscientiousness as well as their interaction term. The main effect of conscientiousness approached to be a significant predictor of error rate, *F*_(1,26)_ = 3.13, *p* = 0.09, whereas other effects remained non-significant, *F*s < 1.40, *p*s > 0.26. The analysis of RTs showed that the main effect of rule condition was significant, *F*_(1,52)_ = 5.97, *p* = 0.005 and other effects remained non-significant, *F*s < 1.69, *p*s > 0.20. Bonferroni *post hoc* tests used to break down the main effect of rule condition revealed that instruction from STD condition which is the frequent one evoked faster responses than instructions from RR (*p* < 0.001) and RV (*p* < 0.001).

As a last component analyzing the predictive ability of conscientiousness on behavioral performance, we ran a simple linear regression analysis with regressing PES onto the conscientiousness values. The main effect of conscientiousness was non-significant, *β* = −0.22, *t*_(26)_ = 1.12, *p* = 0.27.

### ERPs

#### ERN/CRN

We constructed a linear mixed-effects regression model to examine the influence of rule condition, conscientiousness and accuracy on the ERN/CRN amplitude. As fixed effects, we entered rule condition, conscientiousness and accuracy as well as all possible interaction terms. We found a significant main effect of accuracy on the response-locked ERP within 20–70 ms post-RT interval, *F*_(1,78)_ = 11.25, *p* = 0.001, with higher amplitude on error trials than on correct trials. We also found a significant interaction of conscientiousness and accuracy, *F*_(1,78)_ = 4.46, *p* = 0.038. All other effects remained non-significant, *F*s < 1.02, *p*s > 0.36.

**Figure 4:**
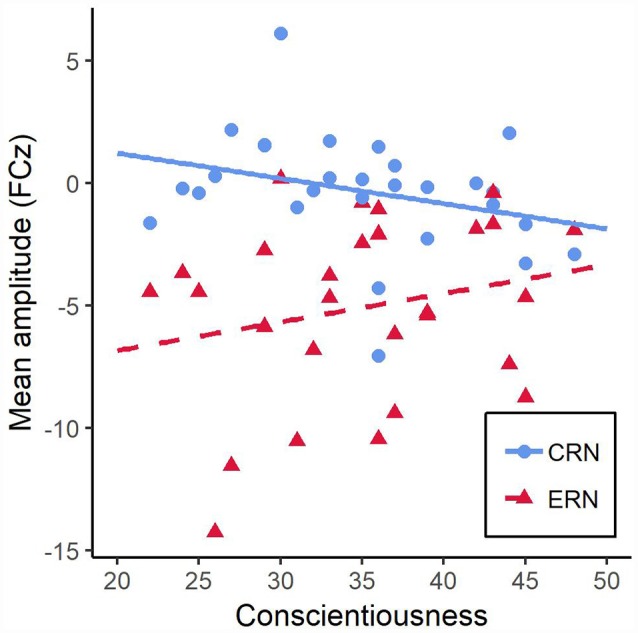
Mean amplitudes during 20–70 ms post-response interval at electrode FCz as a function of conscientiousness for correct (CRN) and incorrect responses error-related negativity (ERN). The lines resulted from regressing CRN and ERN amplitudes onto conscientiousness.

To elucidate the significant interaction of conscientiousness and accuracy, we ran two separate regression analyses to examine the influence of conscientiousness on both the ERN and CRN amplitude. Both regression analyses did not find conscientiousness to be a significant predictor of neither ERN, *β* = 0.22, *t*_(26)_ = 1.16, *p* = 0.26, nor CRN amplitude, *β* = −0.30, *t*_(26)_ = 1.62, *p* = 0.12. However, the regression lines depicted in Figure 4 indicated that higher values of conscientiousness were associated with higher CRN and with lower ERN amplitudes while lower values of conscientiousness were associated with lower CRN and higher ERN amplitudes. Additionally, a regression analyses examining the influence of conscientiousness on ΔERN, the difference score of ERN and CRN amptlitudes, find conscientiousness to be a significant predictor, *β* = 0.38, *t*_(26)_ = 2.10, *p* = 0.046.

#### Pe/Pc

To investigate whether there is an influence of conscientiousness during the rule conditions on the Pe/Pc amplitude, we built a linear mixed-effects regression model with fixed effects rule condition, conscientiousness and accuracy as well as all possible interaction terms. It revealed a significant interaction effect of conscientiousness and accuracy, *F*_(1,78)_ = 5.28, *p* = 0.024. The main effect conscientiousness approached significance, *F*_(1,26)_ = 3.01, *p* = 0.09. All other effects remained non-significant, *F*s < 1.68, *p*s > 0.19.

**Figure 5:**
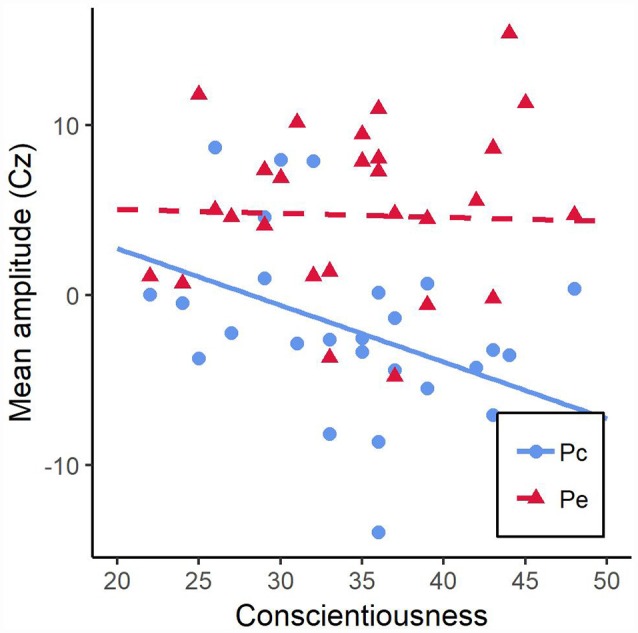
Mean amplitudes during 200–350 ms post-response interval at electrode Cz as a function of conscientiousness for correct (Pc) and incorrect responses (Pe). The lines resulted from regressing Pc and Pe amplitudes onto conscientiousness.

We ran two separate regression analyses examining the influence of conscientiousness on both the Pe and Pc amplitude to elucidate the significant interaction of conscientiousness and accuracy. Conscientiousness was found to be a significant predictor of Pc amplitude, *β* = −0.44, *t*_(26)_ = −2.52, *p* < 0.018, but not of Pe amplitude, *β* = −0.03, *t*_(26)_ = −0.14, *p* = 0.89. The regression lines are depicted in Figure 5. This indicated that lower values of conscientiousness were associated with higher Pc amplitudes.

#### Analysis of CRN and Pc With Larger Sample

To gain more power for the analysis of the CRN, we conducted separate analyses exclusively for CRN and Pc amplitude with a larger dataset including participants that were sorted out before due to insufficient numbers of errors.

Again we conducted a linear mixed-effects regression analysis with fixed effects rule condition and conscientiousness (including interaction term) to examine the influence of conscientiousness during rule conditions on the CRN. The main effect of conscientiousness approached significance, *F*_(1,43)_ = 3.16, *p* = 0.08, whereas other effects remained non-significant, *F*s < 2.14, *p*s > 0.12. These results confirmed the previous indication that there might be an association of conscientiousness and the CRN amplitude.

Another linear mixed-effects regression analysis with fixed effects rule condition and conscientiousness (including interaction term) was computed to investigate whether there was an influence of conscientiousness and rule instructions on the Pc amplitude. A significant main effect of conscientiousness was revealed, *F*_(1,43)_ = 5.51, *p* = 0.024. The main effect of rule condition was found to be significant, *F*_(2,86)_ = 3.79, *p* = 0.027. Bonferroni *post hoc* tests used to break down this main effect of rule condition revealed that instruction from STD condition evoked a lower Pc amplitude than instructions from RR (*p* < 0.001) and RV (*p* < 0.001).

**Figure 6:**
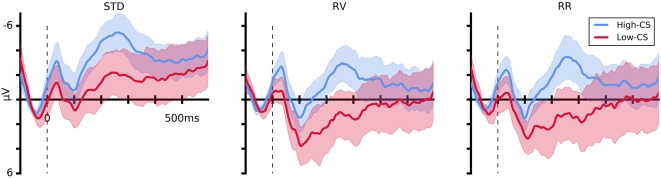
Response-locked event-related potentials (ERPs) for correct responses at electrode FCz for both conscientiousness (CS) groups separated by instruction conditions. The three panels show the ERPs of standard (STD), rule violation (RV), and rule reversal (RR) conditions, respectively. Color-shaded areas indicate the 95% confidence intervals for the mean ERPs. Gray-shaded areas mark the time windows of CRN and Pc.

**Figure 7:**
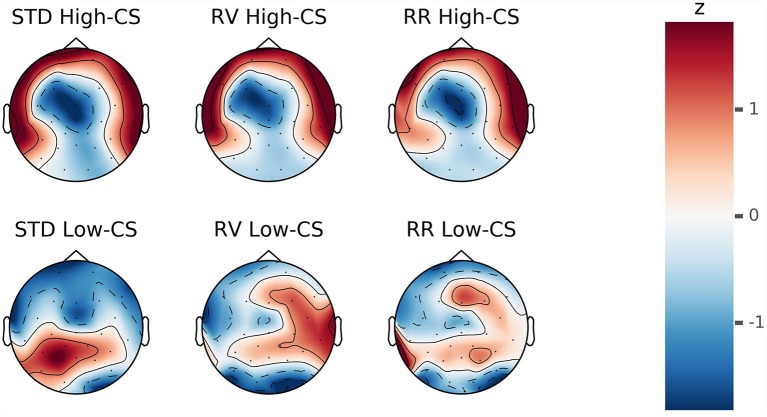
Z-transformed voltage distributions during response-locked ERP after correct responses in a time window of 30–50 ms. The upper and lower heads show distributions of participants scoring high and low on conscientiousness (CS), respectively. The distributions of each group are displayed separately by column for the instruction conditions standard (STD), rule violation (RV) and rule reversal (RR).

**Figure 8:**
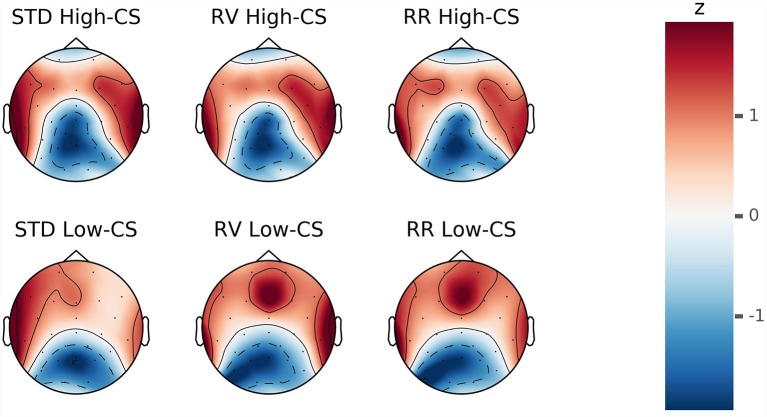
Z-transformed voltage distributions during response-locked ERP after correct responses in a time window of 200–300 ms. The upper and lower heads show distributions of participants scoring high and low on conscientiousness (CS), respectively. The distributions of each group are displayed separately by column for the instruction conditions standard (STD), rule violation (RV) and rule reversal (RR).

For the purpose of illustration, low (*M* = 29.59, *SD* = 4.12) and high (*M* = 41, *SD* = 3.79) conscientiousness groups were formed using a median split procedure. The response-locked ERP averages of trials with correct responses separated by conscientiousness groups (low or high) are depicted in Figure 6. Topographical maps of CRN and Pc are illustrated in Figures 7, 8.

## Discussion

The main purpose of this experiment was to examine the variability of ERN and CRN-amplitude as a function of conscientiousness and rule violation behavior. For this purpose, we designed a choice reaction task with simple rules consisting of responding to a circle and a square pressing the left and the right key, respectively. In two other conditions, participants were asked to either reverse or violate the instructed rules. We assumed that conscientiousness is associated with a better task performance (displayed by lower error rates and shortened RTs) but higher cognitive conflict (displayed by prolonged PES) as well as increased ERN and CRN. Rule violations were expected to be accompanied by prolonged RTs and an increase in ERN and CRN.

The results of the present study partly confirmed our hypotheses. There are weak signs of better task performance for more conscientious individuals which manifested in a tendency of lower error rates but not shorter RTs. We observed an electrophysiological signature that was associated with conscientiousness. An interaction of conscientiousness and accuracy showed that the ΔERN was negatively related to conscientiousness. This pattern descriptively manifested by both a positive relationship of the CRN magnitude with conscientiousness and a negative relationship of the ERN amplitude with conscientiousness. Electrophysiological differences were also revealed by an association of conscientiousness and the magnitude of the Pc. Lower values on the conscientiousness scale were associated with higher amplitudes of the Pc. In addition, comparing RV and RR trials to STD trials, participants were showing a lower amplitude of CRN and a more pronounced amplitude of Pc. Opposed to our hypotheses, the instruction to violate the rules had the same effect as simple task switches on both behavioral performance and response-locked ERPs at medial-frontal electrodes. There was also no measurable influence of conscientiousness on these effects.

It should be noted that all findings regarding the ERN should be interpreted with caution due to the relatively small number of errors generated overall, *M* = 5.5%, *SE* = 0.38. This might be a result of our experimental design. After artifact rejection only few trials were left for analysis of variations in ERN amplitude. We only included participants with at least five error trials in our analyses (except for particular analyses concerning CRN/Pc where only correct trials were needed). However, it is recommended to have a minimal trial number of 15 for within-subject comparisons (Fischer et al., 2017). Future research may aim at altering the experimental design so that participants are forced to commit more errors.

### Higher Task Engagement in Conscientious Individuals

The weak association of lower error rates and high conscientiousness might indicate a different task performance strategy which may be affected by a greater concern over errors and a higher motivation to respond correctly. However, this interpretation is rather speculative since there was only the tendency of lower error rates in high conscientious individuals. This result pattern might indicate that participants scoring high on the conscientiousness scale made a greater effort to respond correctly in contrast to individuals with lower levels of conscientiousness. More conscientious individuals may, therefore, have a stronger focus on task performance.

The ERP findings would support the interpretations drawn from the behavioral data concerning task performance strategies. Most intriguingly, there was an inverse relationship of the amplitudes of ERN and CRN depending on conscientiousness. With an increase of conscientiousness, the amplitude of ERN decreased while the magnitude of CRN enhanced. This was also expressed in a decrease of the ΔERN when conscientiousness increased. This observation in combination with better accuracy values of more conscientious individuals is in line with the assumption that CRN reflects task engagement (Simons, 2010). It also supports the notion that individuals with high values on the conscientiousness scale are characterized by stronger task engagement and stronger focus on task performance. Additionally, for participants high in conscientiousness, the indicated pattern of enhanced CRN amplitude associated with lower error rates is hinting in the same direction as findings that CRN magnitude is inversely related to the probability of committing errors (Allain et al., 2004). Allain et al. (2004) believe that larger CRN amplitudes could have a preventive function against committing errors. Moreover, some other studies reported similar patterns providing further evidence for the interpretation of different task performance strategies. For example, a recent study of Kałamała et al. (2018) found enhanced ERN and CRN amplitudes as well as higher accuracy values in bilinguals suggesting that they are pursuing an accuracy-focused strategy. Hajcak and Simons (2002) also reported enhanced ERN and CRN in participants with obsessive-compulsive characteristics.

We, therefore, conclude that conscientiousness is associated with a heightened motivational salience of an error-free task completion. Following the idea that ERN is reflecting error significance (Hajcak et al., 2005a), the CRN may reflect a “correct response significance” and we suggest that higher values of conscientiousness may be linked to a stronger motivation to correspond correctly due to the absence of a more pronounced ERN.

There might be a possible confoundation with anxiety, especially apprehension/worry which is related to higher ERN amplitudes (for a review, see Moser et al., 2013) and cannot be cleared in our study due to a missing control for this trait. However, according to Moser et al. (2013), there is no clear relationship of anxiety with higher magnitudes of CRN. In contrast, our data suggest that conscientiousness is related to lower or equal ERN but a tendency to higher CRN amplitudes. Thus, the electrophysiological pattern related to conscientiousness seems to contradict the pattern related to anxiety.

In addition to a CRN that is tended to be increased in conscientious participants, there was an association of reduced Pc amplitudes and high conscientious individuals. In past studies, trials preceding errors were found to be characterized by a similar positivity following the CRN (Allain et al., 2004; Hajcak et al., 2005b). It was interpreted as disengagement of the ACC from the monitoring process (Ridderinkhof et al., 2003). Although our analysis was not exclusively concerned with trials preceding errors, the increased Pc in participants with low levels of conscientiousness in our study could also be explained by a disengagement of the response monitoring process. This view was also supported by higher error rates in participants scoring low on the conscientiousness scale. A Pc has also been found in participants with high negative affect (Hajcak et al., 2004). Hajcak et al. (2004) found that this post-response positivity was more pronounced in an infrequent task. In line with their observation, we also found that the Pc was more pronounced in the RV and RR conditions (the infrequent conditions). The Pc might reflect the expectation of the upcoming trial type or a preparation process that controls how to respond next. To examine this assumption, future experimental settings have to investigate the role of frequency of the trial types.

It is conceivable that the observed Pc is similar to the post-response positive activity after errors known as Pe usually occurring during an interval of 200–350 ms following the erroneous response. According to the assumption that ERN and CRN represent in part an identical cognitive control process during response monitoring (Suchan et al., 2007; Burle et al., 2008; Meckler et al., 2011), Pe and Pc could both be associated with an identical process as well. With regard to errors, there are two approaches to explain the Pe. It could either reflect error awareness (Overbeek et al., 2005; Steinhauser and Yeung, 2010) or confidence about response correctness (Boldt and Yeung, 2015). Boldt and Yeung found that confidence about response correctness varied inversely as a function of Pe magnitude. According to the last perspective and transferred to correct responses, our data indicate that individuals with low levels of conscientiousness are less confident about the response-correctness which could also be explained by task disengagement.

### Effects of Rule Violations

We observed generally higher RTs and error rates in RV and RR trials indicating a greater response conflict in these conditions. The findings concerning rule violations are partly in line with previous research on rule violation behavior (Pfister et al., 2016b; Wirth et al., 2016). However, RTs and error rates from our study did not differ between rule violations and rule reversals which may imply that violating a rule does not differ from changing the rule with regard to the evoked cognitive conflict. The nonexistent effects of rule violations in our study might be due to the within-subject manipulation we chose while effects found by Wirth et al. (2016) were based on a between-group design. Corresponding to the nonexistent effects of rule violations in behavioral data, we did not observe any differences in the response-locked ERPs between rule violations and rule reversals which also might be due to our experimental design.

The behaviorally observed cognitive conflict during RV and RR trials (compared to STD trials) could be attributed to a concurrent activation of multiple competing responses. We argued that such a cognitive conflict could have an influence on the ERN or CRN amplitudes. However, according to our observations, this conflict was not reflected by differences in ERN, supporting evidence that questions conflict monitoring approaches (Carbonnell and Falkenstein, 2006; Masaki et al., 2007; Burle et al., 2008). On the contrary, there were equal CRN amplitudes in high conflict trials from RV and RR conditions compared to low conflict trials from STD condition although arguing from a conflict monitoring perspective one could expect higher CRN amplitudes.

A Pc-like component in correct rule violations was at least descriptively also found in the study of Pfister et al. (2016a) but the authors left it without comment. However, our data suggest that the similarity of the waveform in both RV and RR trials indicates that this component may reflect a more general process related to rule modifications, not one specific to rule violations.

### Problems With Our Task Design

Although our task could highlight some differences in performance monitoring of conscientious individuals, it seems obvious that our experimental manipulation failed with respect to the initiation of rule violation behavior. Possible explanations could be the following. First, some participants reported that they internally translated instructions from both RV and RR condition to one instruction that required the same response. They obviously reframed the instructions and did not differentiate the meaning of violating the rule from reversing the rule. Second, a habituation effect could have occurred when violating the rule was instructed too often since 20% of all trials were rule violation trials. Lastly, the instruction to violate a rule may not have created an ecologically valid feeling of breaking a rule in participants at all. The explicit instruction to violate the rule might have had the effect of a legitimization to break the rule leading to the feeling of rule-compliant behavior instead.

These observations as well as the low error rate mentioned earlier showed that our task was less than ideal. There are some crucial steps to improve the task. First, the implementation of an Eriksen flanker task (Eriksen and Eriksen, 1974) might lead to a higher error rate. In contrast to the simple choice-reaction task we chose, the flanker task has been successfully utilized to achieve a suitable number of errors. Second, the experimental manipulation of rule violations should be based on a between-group design. Instead of instructing to violate and to reverse the rule in the same participant, both instructions should be given in different groups to prevent reframing of task instructions. These alterations in the task design may shed light upon whether there are effects of rule violations in choice-reaction tasks associated with conscientiousness.

### Limitations

It has to be mentioned that all findings regarding conscientiousness should be interpreted with caution due to the distribution of conscientiousness values in our sample. The whole sample actually exhibited conscientiousness values above average corresponding to actual moderate to high conscientiousness values. Future research should aim at observing response monitoring after obtaining a broader range of conscientiousness values.

### Conclusion

To summarize, the present study provided the first evidence that conscientiousness might be associated with the ΔERN amplitude. Our data indicate that high conscientiousness is linked to a larger magnitude of the CRN and smaller amplitude of the ERN. The decrease of ΔERN with an increase in conscientiousness might be explained by a shift of motivational significance of errors towards a motivational significance of correct responses. This supports the perspective that increases in CRN amplitude might reflect task engagement and motivational salience of an error-free task completion, which can also be expressed as “correct-response significance.” This perspective is further strengthened by higher Pc amplitudes in individuals having low levels of conscientiousness which has been linked to disengagement of the response monitoring. Furthermore, cognitive conflict during rule violations and rule reversals indicated by prolonged RTs and higher error rates was not reflected by differences in CRN or ERN. This observation contradicted conflict model accounts of the ERN.

## Ethics Statement

All participants signed a consent form prior to participation in the study. The study was conducted in accordance with the Declaration of Helsinki. The ethics committee of the University of Bamberg approved the study protocol.

## Author Contributions

JR and MI designed the study, wrote the article and approved the final manuscript. MI collected and analyzed the data.

## Conflict of Interest Statement

The authors declare that the research was conducted in the absence of any commercial or financial relationships that could be construed as a potential conflict of interest.
